# The Danger of Dust as a Cause of Tuberculosis

**Published:** 1907-05

**Authors:** 


### THE DANGER OF DUST AS A CAUSE OF TUBERCULOSIS.

George Homan, in discussing insanitary methods used in cleaning public places, says:

Efforts toward the eradication of human tuberculosis will fail which do not take full account of household dust as a factor in the dissemination of that disease.

Scientific tests have shown that the seeds of pulmonary tuberculosis, harbored within doors in the dried state, are capable of retaining their effective vitality for prolonged periods of time.

Any method or procedure employed in inhabitated buildings which causes dust to be disseminated must be considered as tending to spread the seeds of consumption.

Hotels, clubs, theaters, office buildings, schools, churches and business etablishments generally should be required by law to introduce and operate dustless methods of cleaning; this part of their mechanical equipment as necessary as pkrovisions similarly made for warming, ventilation and for fire protection and fire escape. The employment of dustless methods in private residences is urged as being equally imperative for the control and suppression of all forms of tuberculosis disease.—Journal A. M. A., March 23, 1907.

